# Editorial: Home cage-based phenotyping in rodents: innovation, standardization, reproducibility, and translational improvement, volume II

**DOI:** 10.3389/fnins.2026.1894837

**Published:** 2026-07-02

**Authors:** Lars Lewejohann, Oliver Stiedl, Stefano Gaburro, Silvia Mandillo

**Affiliations:** 1 German Federal Institute for Risk Assessment (BfR), German Centre for the Protection of Laboratory Animals (Bf3R), Berlin, Germany; 2 Institute of Animal Welfare, Animal Behavior and Laboratory Animal Science, Freie Universität Berlin, Berlin, Germany; 3 Animal Welfare Body, Amsterdam University Medical Center (Amsterdam UMC) and Vrije Universiteit, Amsterdam, Netherlands; 4 Digital Preclinical Society, Cambridge, MA, United States; 5 Independent Researcher, Berlin, Germany; 6 Institute of Biochemistry and Cell Biology (IBBC), National Research Council (CNR), Monterotondo, Italy

**Keywords:** animal welfare, data analysis, disease model, home cage monitoring, mouse behavior

Behavioral neuroscience has relied for decades on test paradigms that remove animals from their home cage, place them in novel arenas, and record responses commonly only for a few minutes. The logic seems self-evident: controlled conditions produce controlled data. Yet, the assumption deserves scrutiny. Novel environments and handling procedures are stressors triggering neuroendocrine responses. Short observation windows sample only a fraction of the circadian cycle. Experimenter presence introduces variability that is difficult to standardize across laboratories and across experimenters within the same laboratory (Crabbe et al., 1999; Lewejohann et al., 2006). What we gain in experimental control we often pay for in external validity, ethological and ecological relevance, replicability and, ultimately, in translatability.

Home cage monitoring (HCM) takes a different approach. Instead of bringing the animal to the test, HCM brings the test to the animal. Rodents are monitored continuously in the cage where they live, eat, sleep, and interact. Data collection spans the full light-dark cycle, over days or weeks, without handling, transport, or habituation to novel environments. This is not merely a technical convenience. It changes the nature of the behavioral signal from an exceptional situation to normal behavior. Continuous recording captures circadian structure, inter-individual variability, and slow-onset phenotypes that a 10-mins open field test might not resolve. On the other hand, when adopting this approach, we must ask ourselves in particular whether the lives of our laboratory animals in small, standardized cages are representative of the biology of the species in question. In fact, it has repeatedly been shown that HCM is not limited to small, minimally stimulating enclosures. Its full potential can likewise be exploited in more complex enclosures, right up to semi-natural environments in which a home cage can be connected to different accessible environments (Mieske et al., 2021; Rivalan et al.). Now the animal deliberately decides if and when to explore these environments.

This is already the second volume of the research topics series (see Gaburro et al., 2022) devoted to the fascinating and highly timely topic of HCM in laboratory rodents. The foundations for both editions were laid in the highly successful COST Action “TEATIME” (a humorous acronym for “improving biomedical research by auTomated bEhAviour moniTorIng in the aniMal home-cagE,” as we usually met at teatime). This field of research had already shown great promise even before the launch of TEATIME. During the 4 years of EU funding for the COST action, the topic gained tremendous significance, driven by the enthusiasm of an active, creative, dynamic, and highly connected group of researchers. It quickly became clear that a single Research Topic in Frontiers would not be nearly enough, which is why we are delighted to present this second Research Topic. The eight articles assembled in this Research Topic illustrate what is feasible when behavioral data are collected using HCM. We would like to thank all the authors for their valuable contributions to this volume.

Khatiz et al. monitored C57BL/6J mice continuously through the oestrus cycle and found that females in oestrus showed up to 30% higher locomotor activity than males, while males exhibited higher sleep-related and habituation behaviors across all cycle phases. These sex- and phase-dependent differences were not detected in earlier studies relying on single time-point assessments. This study provides valuable insights into the link between behavioral differences related to sex, reproductive cycle, and circadian rhythm.

Tomanelli et al. reported activity effects of conditional KRAS and KRAS-LKB1 transgenic mice as lung cancer models since KRAS mutation occurs in 25% of all lung cancers. The concomitant mutations in LKB1 determine aggressive subtypes of these tumors. KRAS and KRAS-LKB1 mutations were induced, and the tumor formation and progression were monitored over time (MRI) and locomotor activity was monitored in Digital Ventilated Cages (DVCs). After tumor induction by Cre-recombinase, with earlier tumor onset in KRAS-LKB1 than in KRAS transgenic mouse line, the locomotor activity decline was more pronounced in the dark than in the light phase and correlated with the tumor growth determined by MRI measurements. These experiments show activity monitoring in the home cage can play an important role in early onset detection of disease symptoms and its progression from a scientific viewpoint and can also be used with objective criteria to apply humane endpoints to avoid unnecessary discomfort as diseases progresses and determine effective therapeutic interventions more efficiently.

Moore et al. were also using the DVC system and followed an aging colony of C57BL/6 mice for 18 months. Long term observations of laboratory mice are indeed very rare in the literature and following a colony over such a long time resembles an extraordinarily rich data set. Activity decreased progressively between 5 and 14 months, then partially recovered. More fine graded differences in activity were revealed related to weekly cage cleaning. This is certainly not unexpected, but it does provide a sound basis for future studies to adapt their experimental design to the cleaning cycle. The same dataset flagged stereotypic behaviors through sustained activity spikes, opening a welfare monitoring application that conventional cage-side scoring would miss at scale.

Sun et al. validated DVC-derived circadian activity profiles of group-housed mice against an established activity measurement system. Both systems can detect genotype and treatment effects effectively, despite technological differences (EMF-based vs. I.R beam frame). After standardizing the data using *Z*-scores, the profiles were reproducible, scientifically accurate, and confirmed both a hyperactivity phenotype in an Alzheimer's disease model and an activity reduction in a type-1-like diabetes model. That these results come from group-housed animals is important: individual housing alters behavior profoundly, and any monitoring approach that requires isolation introduces a confound at the level of the housing condition itself.

Rivalan et al. placed serotonin-deficient Tph2 knockout mice in a visible burrow system and used machine learning to classify behavioral time series. Allogrooming, feeder struggles, and eating emerged as the features that best separated genotypes. Social network analysis revealed that pathological aggression in individual knockout animals disrupted the formation of stable group hierarchies, a finding with direct relevance for modeling psychiatric comorbidity of uncontrolled aggression.

Abdollahi Nejat et al. used DVC-based locomotor monitoring to evaluate welfare after intracranial stereotaxic surgery in APP/PS1 mice serving as a model of Alzheimer's disease. They show significantly reduced locomotor activity, with incomplete recovery even after six days, while circadian rhythms remain stable and body weight changes are minimal or age-dependent. These findings highlight that continuous monitoring of locomotor activity is a more sensitive indicator of post-surgical impact than body weight and emphasize the need for extended recovery periods before experiments. In one case, a mouse with an aberrant activity spike died suddenly, possibly from epileptic activity (sudden death in epilepsy; SUDEP) known in this mouse strain as breeding line with discomfort. This is individualized welfare assessment, operating at a resolution that cage-side observation cannot match. Overall, the work underscores the importance of improved monitoring, analgesic protocols, and direct seizure detection to enhance animal welfare and for an effective severity assessment as required for new genetic lines to determine potential discomfort.

Correia et al. benchmarked three pipelines for automated grooming quantification: DeepLabCut/SimBA, HomeCageScan, and manual scoring. DeepLabCut/SimBA correlated well with manual scoring for grooming duration but diverged at the level of bout detection. HomeCageScan overestimated duration at low grooming levels. These discrepancies matter. As HCM generates increasingly large datasets, the temptation to trust automated classifiers without validation against ground truth will grow. This paper is a necessary corrective because there is a growing need to determine the best tools for behavior analysis for optimally standardized, quantitative approaches for improved comparability and replicability in science.

Golini et al. reported a profoundly increased rest time during the night (active) phase of DMSXL mice carrying a mutated human DMPK transgene containing >1,000 CTG repeats. This genetically modified mouse strain is a model of a severe early onset form of myotonic dystrophy type 1 (DM1), a dominantly inherited neuromuscular disease caused by the abnormal expansion of CTG-repeats in the 3′-untranslated region of the dystrophia myotonica protein kinase (DMPK). Circadian activity measurements were performed in pairs of mice housed in DVCs showed excessive rest during the active phase and disrupted regularity indices when monitored in over 5 weeks. The phenotype mirrors excessive daytime sleepiness reported in DM1 patients and was detected entirely non-invasively, providing a translational readout for therapeutic screening.

What connects these eight studies is not only the use of HCM technology but a shared methodological commitment: continuous data collection from undisturbed animals in their living environment. This commitment produces converging insights across very different biological questions starting out with the effects of estrous cycle (Khatiz et al.) via disease progression in cancer and neurodegeneration (Tomanelli et al., Sun et al. and Golini et al.) manifested as altered circadian locomotor patterns. Post-surgical welfare deterioration (Abdollahi Nejat et al.) and age-related behavioral change (Moore et al.) also affect activity patterns and thus can be monitored using HCM technology. Evaluating the most appropriate analytical tools is a critical issue. Ultimately, selecting the most suitable systems could be vital for future standardization (Correia et al.). However, we must also recognize that we are operating in a dynamic scientific field where technical advances are rapidly evolving. It is therefore less a matter of identifying a tool that is suitable for all time, but rather of establishing a framework and arriving at well-coordinated definitions that allow for comparability across various current and future technologies. It is important to note that welfare monitoring and scientific phenotyping are not independent parallel tracks. They share the same data stream. This convergence is not just a convenient coincidence. It reflects the biological fact that compromised welfare corrupts experimental readouts. These approaches allow to better define the physiology-to-pathology gap that still exists due to the lack of appropriate long-term monitoring of activity in health and disease that is affected by many variables and influencing factors that play a role here, such as mouse and rat sub-strains, age, sex, food, housing conditions, microbiome, and many more (Potschka et al., 2026).

The development of HCM as a method for various disciplines and the awareness about its important scientific and welfare contribution has been significantly advanced by joint European networking initiatives. As mentioned above, this has been achieved in particular through the COST Action CA20135 (TEATIME, www.cost-teatime.org). Founded by 58 researchers across 23 countries, TEATIME has built a pan-European network of behavioral scientists, data scientists, technology developers, and animal welfare specialists working toward common protocols, shared baseline datasets, and cross-comparable analytical frameworks. Its five working groups address community coordination, technology assessment, bioinformatics and data integration, training, and dissemination. Several contributions to this Research Topic originate from laboratories connected through TEATIME, and the network's emphasis on bridging behavioral and data science has shaped the analytical approaches visible across these papers. Even after the funding period has ended, the network remains of crucial importance. All researchers working in the field of HCM are kindly invited to check the Research Topic of resources available at the TEATIME website (https://www.cost-teatime.org/) and get involved through interactive platforms such as The Behavior Forum (https://www.thebehaviourforum.org/).

In addition to these past and future joint efforts, the first comprehensive reference work on the subject of HCM has just been published: “Home Cage Monitoring in Rodents: A Global Effort” (Gaburro and Mandillo, 2026), an Open Access book prepared by more than 60 authors from across the field. The volume covers animal welfare aspects, recommendations for DIY systems, training, available technologies, rodent models, study design, commercialization pathways, and data analysis and sharing. It is designed to serve as a practical guide for laboratories entering the field and as a reference for those already applying HCM in their research programs. As such, the book is the most comprehensive reference work in the field, and one that goes to great lengths to give back knowledge and enthusiasm to this wonderful community.

Where does HCM go from here? Whilst the legal framework governing animal testing is generally becoming more complex, these regulations are actually encouraging the use and further development of HCM systems. Both the “FDA Modernization Act 2.0” and the European regulatory focus on the 3Rs are steering research toward data-rich, animal-welfare-oriented experimental approaches. But technology alone does not solve the problem. Standardized metadata, FAIR data pipelines, validated behavioral ontologies, and transparent reporting of classifier performance are prerequisites for scaling HCM from individual laboratory installations to a field-wide analytical infrastructure. The analytical diversity across the papers in this Topic (principal component analysis, linear mixed models, machine learning classifiers, social network analysis) reflects a field still assembling its methodological toolkit. This task would benefit from coordination and calls for shared standards.

The eight studies collected here represent an additional and updated cross-section of what HCM enables today. The range is already broad: from reproductive biology to oncology, from neurodegenerative disease to psychiatric modeling, from welfare assessment to analytical validation. Continuous monitoring of rodents in the home cage is not an incremental improvement on existing methods. It is a different experimental paradigm, one that aligns scientific rigor with animal welfare rather than trading one for the other as future gold standard in behavioral neuroscience. The work presented in this Research Topic shows that the community building this paradigm is international, methodologically diverse, and growing. The network is taking shape and continues to grow, and the promising developments in this area suggest that the paradigm shift from short-term testing to monitoring in the home cage will continue to take hold.
